# Supplementary material for: The interactome of intact mitochondria by cross-linking mass spectrometry provides evidence for coexisting respiratory supercomplexes
Source: Mol Cell Proteomics. 2017 Dec 8;17(2):216–32. doi: 10.1074/mcp.RA117.000470 (PMC5795388; doi:10.1074/mcp.RA117.000470)
Supplement: Supplemental Data [file supp_RA117.000470_133922_0_supp_23978_fzffwf.zip › spectra_annotation/mito_spectra_annotation/211-1-4-1-2-1.pdf]

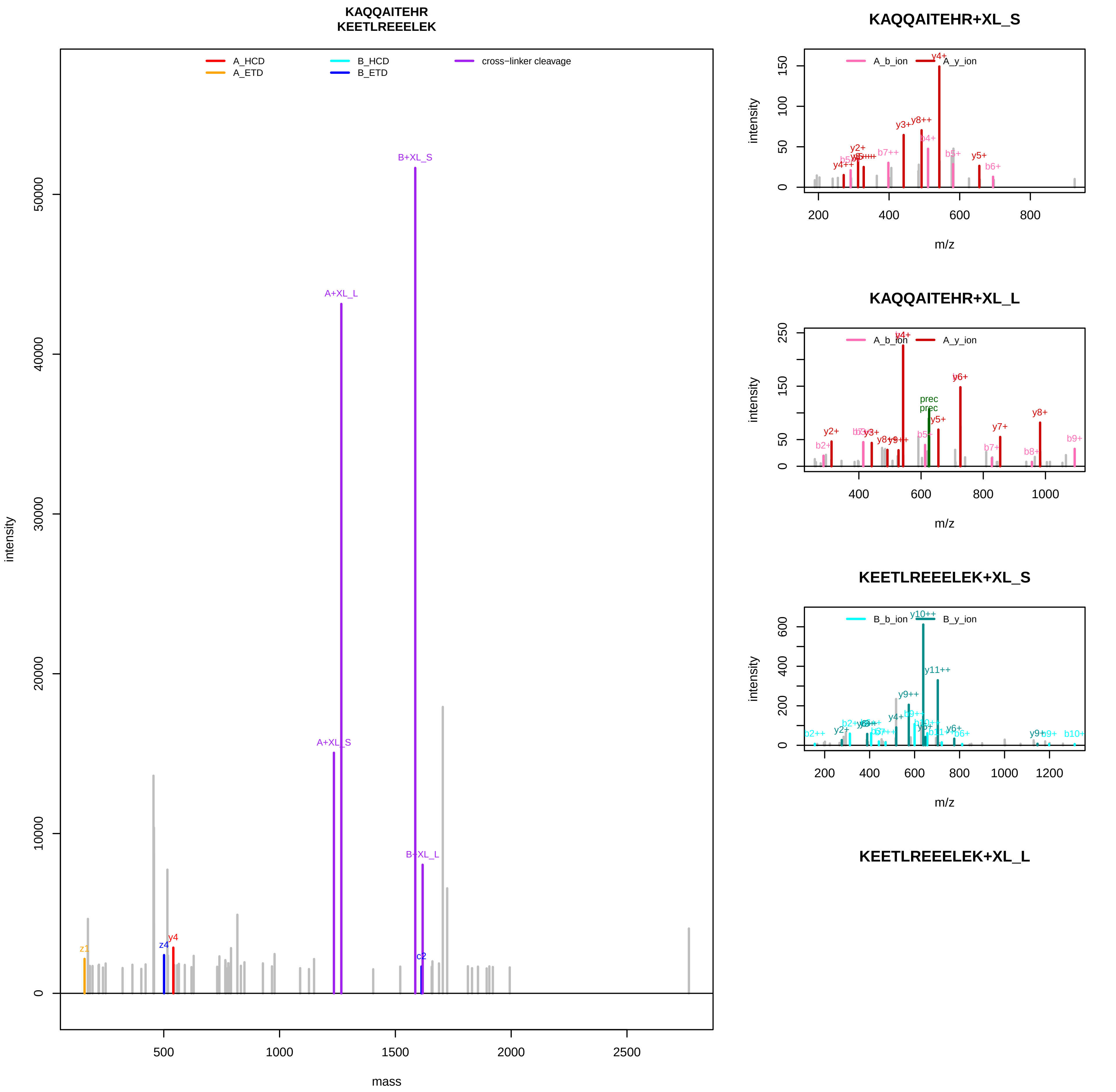
KAQQAITEHR
KEETLREEELEK
A_HCD
A_ETD
B_HCD
B_ETD
cross−linker cleavage
B+XL_S
A+XL_L
A+XL_S
B+XL_L
z1
z4
y4
c2
intensity
mass
0
10000
20000
30000
40000
50000
500
1000
1500
2000
2500
KAQQAITEHR+XL_S
A_b_ion
A_y_ion
y4+
y3+
y8++
y2+
b7++
b4+
b5+
y5+
b6+
y4++
m/z
KAQQAITEHR+XL_L
y6+
y8+
y7+
y5+
y3+
y2+
b2+
b9+
b8+
b7+
prec
KEETLREEELEK+XL_S
B_b_ion
B_y_ion
y10++
y11++
y9++
y4+
y6+
y9+
b9+
b10+
b2++
y2+
KEETLREEELEK+XL_L
